# Utilizing Health Behavior Change and Technology Acceptance Models to Predict the Adoption of COVID-19 Contact Tracing Apps: Cross-sectional Survey Study

**DOI:** 10.2196/25447

**Published:** 2021-05-19

**Authors:** Samuel Tomczyk, Simon Barth, Silke Schmidt, Holger Muehlan

**Affiliations:** 1 Department Health and Prevention Institute of Psychology University of Greifswald Greifswald Germany

**Keywords:** mHealth, COVID-19, UTAUT1, UTAUT2, health behavior change, theory of planned behavior, contact tracing, app, model, technology acceptance, cross-sectional studies, social norms, health communication, privacy, anxiety

## Abstract

**Background:**

To combat the global COVID-19 pandemic, contact tracing apps have been discussed as digital health solutions to track infection chains and provide appropriate information. However, observational studies point to low acceptance in most countries, and few studies have yet examined theory-based predictors of app use in the general population to guide health communication efforts.

**Objective:**

This study utilizes established health behavior change and technology acceptance models to predict adoption intentions and frequency of current app use.

**Methods:**

We conducted a cross-sectional online survey between May and July 2020 in a German convenience sample (N=349; mean age 35.62 years; n=226, 65.3% female). To inspect the incremental validity of model constructs as well as additional variables (privacy concerns, personalization), hierarchical regression models were applied, controlling for covariates.

**Results:**

The theory of planned behavior and the unified theory of acceptance and use of technology predicted adoption intentions (R^2^=56%-63%) and frequency of current app use (R^2^=33%-37%). A combined model only marginally increased the predictive value by about 5%, but lower privacy concerns and higher threat appraisals (ie, anticipatory anxiety) significantly predicted app use when included as additional variables. Moreover, the impact of perceived usefulness was positive for adoption intentions but negative for frequency of current app use.

**Conclusions:**

This study identified several theory-based predictors of contact tracing app use. However, few constructs, such as social norms, have a consistent positive effect across models and outcomes. Further research is required to replicate these observations, and to examine the interconnectedness of these constructs and their impact throughout the pandemic. Nevertheless, the findings suggest that promulgating affirmative social norms and positive emotional effects of app use, as well as addressing health concerns, might be promising strategies to foster adoption intentions and app use in the general population.

## Introduction

### Background

With the global spread of the COVID-19 pandemic, there have been numerous efforts to develop digital and mobile health (mHealth) solutions to combat the spread of infections [1,2], support quarantine and social isolation, and improve monitoring and communication surrounding the virus [3]. To this end, COVID-19 contact tracing apps were proposed as a way to (1) monitor and track infection chains, (2) provide immediate support and information in case of an infection or contact with an infected person, (3) and support persons in quarantine by monitoring health and tailoring information and preventive actions [1,3,4]. Overall, contact tracing via mobile apps aims to increase perceived safety and security of the population, and to contain infections, some apps also include additional educational information and news updates on governmental regulations [5].

However, these developments encounter several ethical and practical challenges (eg, [1,6,7]). From a technical point of view, for instance, flawless performance of an app is essential to avoid false positives and for it to be perceived as accurate, reliable, and trustworthy (ie, efficacious) [8]. Research shows that perceived performance efficacy of a digital technology predicts behavioral compliance when confronted with false-alarm–prone systems [9]; therefore, the rate of false-positive alarms should be as low as possible for tracing apps. Furthermore, the app has to be accessible across different areas and regions (eg, rural and urban areas) to guarantee successful preventive tracing [7]. In addition, ethical questions concerning the digital divide resurface in this context [6,7,10]. Since the digital divide characterizes differences in access and reach of digital technologies (ie, primary divide) as well as capabilities and habit of use (ie, secondary divide), it challenges tracing apps as public health measures [10]. Elderly people, for example, report less use of smartphones, and they feel less competent in smartphone use [11,12]. This is particularly challenging regarding contact tracing apps, as older people represent a risk group for COVID-19 infections with a higher chance of more severe trajectories [13]. Additionally, using tracing apps also implies an agreement to share health-related data (ie, infection status) via mobile apps with governmental institutions or other citizens, raising concerns surrounding data privacy and a potential breach as well as misuse of health information beyond COVID-19 purposes (cf “surveillance creep” [3,6,7]). Since the implementation of the European Union General Data Protection Regulation in 2018, privacy concerns have repeatedly been discussed as a key variable in influencing attitudes and adoption intentions [14], particularly in the context of mHealth apps.

Hence, lively debates among journalists, scientific experts, and policymakers about the importance of weighing privacy, individual data security, and societal public health needs in digitally tracing COVID-19 infections have led to a variety of diverse developments in mHealth (eg, see [5,15]). A review conducted in May 2020 [15] described 17 tracing apps in 15 different countries with varying degrees of data processing and protection—in September 2020, the Council of Europe already listed 52 contact tracing apps worldwide [16]. Yet only 3 of these 17 reviewed apps (COVIDSafe in Australia, The e-Rouska in the Czech Republic, and VirusRadar in Hungary) were protected by respective data protection laws and provided consistent information on data storage and use policies.

In general, tracing apps tend to follow either a centralized or decentralized data processing approach or a hybrid of both, where either governmental or service institutions receive, monitor, and administer app data via a central server structure (eg, in France), or communication relies on peer-to-peer technology such as Bluetooth (eg, in Germany), to exchange randomly generated codes between app users within a certain radius (eg, a proximity of a few meters) to trace contacts. Either way, once a person’s infection is validated by a health agency, a warning can be sent to stored contacts (centralized approach) or recipients of codes (decentralized approach) to inform them of a potential infection. So far, advantages and disadvantages regarding systems architecture, perceived responsibility, security, and privacy have already been discussed, ranging from personal first-hand experience [17] to systematic reviews (eg, [18]). While further differences between these approaches are beyond the scope of this paper and are discussed in detail elsewhere (eg, systems architecture [19,20]), it is important to note that these processes may further affect adoption intentions and continued app use in the population, as they are assumed to be connected to personal attitudes, such as perceived control, and privacy concerns. In fact, Trang et al [21] found that a strong privacy design, as implemented in a decentralized approach, predicted app acceptance and adoption intentions among critics of the tracing app as well as undecided participants who represented a majority of the sample. Similarly, a cross-cultural study also reported privacy and security concerns as important barriers to tracing app use [22].

### Use of Contact Tracing Apps in the General Population

Mindful of these technical and ethical challenges of tracing apps, a general question therefore is whether the public uses these apps as intended. Simulation studies report that at least 56% of a population needs to use an app for it to have a public health impact [4]. However, use rates appear to be much lower in the general population so far (eg, [19,20]), with the differences being discussed in terms of data processing approaches, societal technology acceptance, and institutional or legal commitment. The Corona Warn-App (which uses a decentralized approach) was launched as the official German coronavirus contact tracing app on June 16, 2020. Until May 7, 2021, the app was downloaded 27.5 million times, which accounts for about 33.1% of the general population [23,24]. Given this large discrepancy between needed and currently reported app use, behavioral health research can help to identify predictors of app use and derive recommendations for preventive practice to increase app use and address perceived barriers in the general population. To this extent, this study draws from the literature on health behavior change as well as (mobile) technology acceptance to explore tracing app use in the general population. While we are aware of several studies that inspect barriers or motives pertaining to tracing app use (eg, [21,22]), few empirical studies are theory based in that they utilize established health behavior or technology acceptance models to investigate app use. So far, we have identified 3 studies based on the health belief model (HBM) [25], the protection motivation theory (PMT) [26], and the unified theory of acceptance and use of technology (UTAUT) [27]. While these studies investigated behavior change theories, they lack an assessment of social influence or social norms, even though these aspects are key factors of successful behavioral prevention during pandemics [28,29]. We believe that it is important to acknowledge the impact of social influence on contact tracing app use—as explicated in several health behavior change models.

### Health Behavior Change

**Figure 1:**
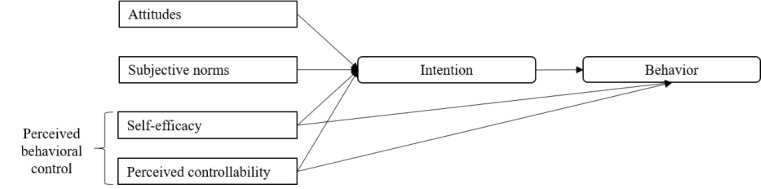
Conceptual model of the theory of planned behavior (adapted from Ajzen [39]).

Ample research provides evidence of psychological processes connected to behavior change toward adaptive health behaviors in pandemics, such as keeping physical distance or practicing personal hygiene [28,30], which have been reiterated during the COVID-19 pandemic (eg, [30-32]). These processes are detailed in health behavior models like the HBM [33], Bandura’s social cognitive theory [34], the PMT [35], and the theory of planned behavior (TPB) [36], which define several constructs associated with behavior change. According to the HBM, for instance, risk perception (eg, susceptibility to and fear of an illness) and perceived benefits (eg, reduced risk of infection) and barriers (eg, high costs, privacy concerns) influence behavior change tendencies. Social cognitive theory further adds the impact of efficacy expectancies [37] that comprise beliefs about the outcome of a behavior (ie, response efficacy, for instance, the belief that wearing masks significantly reduces infection risk) as well as one’s ability to perform within a specific setting (ie, self-efficacy). The PMT combines these approaches by describing threat appraisals (risk perception) as well as coping appraisals (perceived benefits and barriers, response efficacy, and self-efficacy) as predictors of protection motivation and protective behavior. However, applied research points to the intention-behavior gap [38], describing a lack of implementation despite positive intentions, for instance, due to individual forgetfulness or a lack of opportunity to perform. Therefore, the TPB [36] further differentiates efficacy beliefs within the construct of perceived behavioral control (Figure 1).

This construct comprises self-efficacy but also perceived controllability, which refers to the extent to which a person believes to be responsible for their behavioral performance within a specific setting [39]. For example, a person might report high self-efficacy regarding hand hygiene but low controllability due to a lack of soap or disinfectant available to them. Thus, the TPB assumes a direct link between perceived behavioral control and behavior as well as intentions. Other predictors of behavioral intention are attitudes and subjective norms. Attitudes are evaluative judgments of the target behavior, which might include risk perceptions and perceived benefits and barriers, and therefore connect with previously described health behavior models. Subjective norms address descriptive social influence (ie, how many people perform a target behavior) and injunctive social influence (ie, how many people suggest performing a target behavior) on individual intentions. Previous research has cemented the TPB as a popular and versatile framework for predicting health behaviors (eg, [40]) as well as use of mobile apps [41,42]. Current research on the COVID-19 pandemic underlines the predictive validity of the TPB for protective behaviors, with a particularly strong impact of subjective norms and efficacy beliefs [30,43,44]. Similarly, current research on the use of contact tracing apps also finds strong positive associations for response efficacy and moderate positive associations for self-efficacy [25-27]. Subjective norms were only measured in one study [27] and did not show significant associations with app use. However, the measure combined social norms, governmentally provided implementation support, and social support for app use as a composite measure of social influence, resulting in insufficient factorial and content validity.

### Unified Theory of Acceptance and Use of Technology

**Figure 2:**
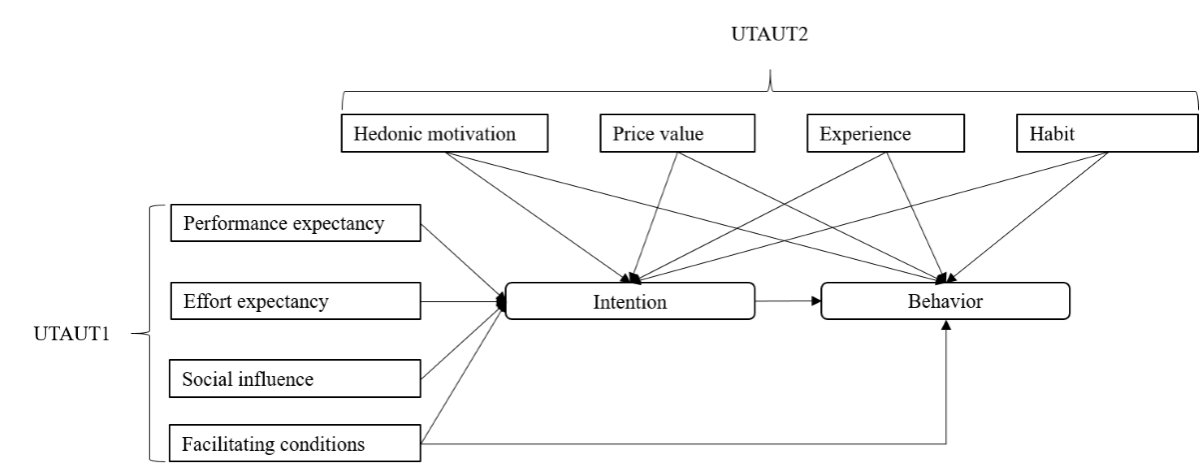
Conceptual model of the unified theory of acceptance and use of technology (UTAUT, first and second iterations) (adapted from Venkatesh et al [50]).

While the TPB has received tremendous attention in health research, its popularity in mHealth research is rivaled by UTAUT [45]. The UTAUT itself is based on the technology acceptance model [46] and the theory of reasoned action [47], a precursor of the TPB, and it also describes psychosocial variables associated with the adoption of (new) technology [48-50]. These variables are performance expectancy, effort expectancy, social influence, and facilitating conditions in the first iteration (UTAUT1), with the addition of hedonic motivation, price value, habit, and experience in the second iteration (UTAUT2) [45,51] (Figure 2).

In contrast to the TPB, however, these descriptions are rather broad, and the model does not set strong theoretical or methodological boundaries for these variables, which has led to many publications but almost as many different iterations of the model, challenging the comparability of findings [50]. Nevertheless, the main implications of the UTAUT are that an adoption of a new technology is more likely if a person expects it to be useful, easy to access and use, recommended and supported by others, and if persons perceive themselves to be able to use it as intended. Moreover, if a person expects positive emotional reactions (eg, joy) and is familiar with similar technology use (ie, habit), this further increases the likelihood. Conversely, disadvantageous facilitating conditions, for example, information privacy concerns or low self-efficacy, can inhibit adoption intentions [25,52].

Conceptually, similarities to health behavior theories are apparent, as expectancies, social influences, and facilitating conditions (eg, self-efficacy, perceived barriers) play an important role in both approaches. Hence, multiple studies have used the UTAUT approach to predict adoption of mHealth apps in general [49], and more specifically, for instance, regarding mobile health care services among the elderly [53], or the use of COVID-19 contact tracing apps [27]. In their study on the acceptance of mHealth services, Sun et al [53] directly compared the predictive value of the TPB, the technology acceptance model (as a reduced version of the UTAUT), the PMT, and an integrated model, and found that an integrated model yields the best results, including positive effects (eg, social influence) and negative effects (eg, threat appraisals) at the same time. Across studies, performance expectancy has the strongest associations with adoption intentions, followed by effort expectancies and social influence. This is in line with current evidence on tracing app use but deviates from health behavior research that reports a similar impact of efficacy beliefs, but a stronger effect of social influence [30,43,44].

Moreover, in their comprehensive meta-analytic review of the UTAUT, Dwivedi et al [48] essentially confirm these observations, but they also point out that most UTAUT studies do not include user attitudes (ie, affective and cognitive evaluations of technology use). Instead, attitudinal assessments are often limited to expectancies. In terms of health behavior theories, this operationalization would exclude aspects of risk perception or threat appraisals (HBM, PMT), and attitudes (TPB), although attitudes were the strongest predictors of behavioral intentions in meta-analytic structural equation models of the UTAUT [48].

Furthermore, the role of social influence (ie, social norms) is not consistently defined in either the health behavior or the technology acceptance approach [54]. In fact, social norms can be categorized as descriptive and injunctive or prescriptive, and further defined as personal or societal, in that they refer to one’s personal surroundings (eg, family or friends) or more general societal categories (eg, persons of the same age, the same country). In short, the reference frame defines the commitment and group orientation, which has been used by nudging interventions based on social identity models to foster technology use [55] or the uptake of vaccinations [56]. Using the general public as a reference can thus shape public behaviors (eg, wearing a mask in public), but does not necessarily affect personal beliefs as strongly (eg, private protective behaviors) [57]. In contrast, personal reference points can increase private behaviors more strongly. In the context of app use, it is therefore important to examine the differential impact of social norms.

### Research Aims

In sum, this study aimed to compare the predictive value of the TPB, the UTAUT, an integrated model, and an extended model (cf [53]) regarding adoption intentions, and current use of COVID-19 infection tracing apps. By exploring both theories separately as well as concurrently, this study provides a blueprint for future research on contact tracing apps and other digital health technologies that combines health-related and technology-related perspectives.

First, we aimed to affirm previous mHealth studies on either the TPB or the UTAUT in the context of COVID-19 contact tracing apps and compared their tenability an integrative model.

Second, while both models have been successfully used to predict health technology use, there are evident shortcomings to both approaches that can be overcome by combining them. Therefore, in this study, we included barriers like privacy concerns, which are more common in technology-focused research (eg, [27,50]), into health behavior models, while also acknowledging the importance of threat appraisals, popularized in health behavior models [53].

**Figure 3:**
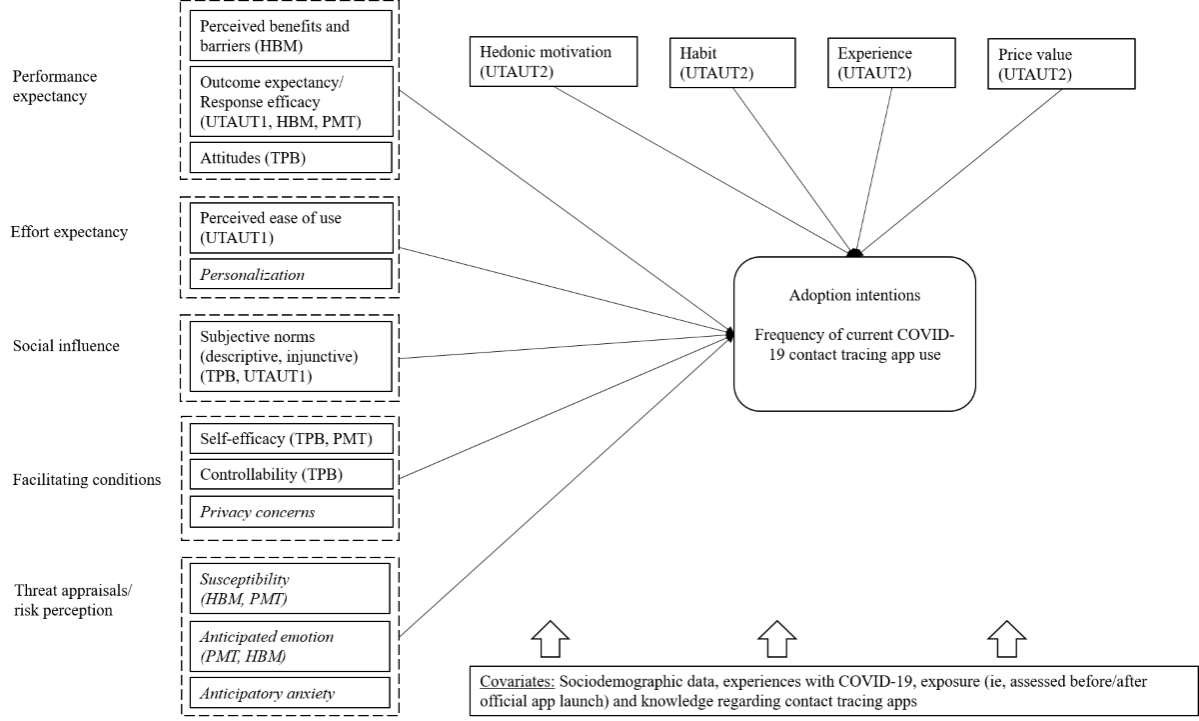
Research framework combining health behavior theories (health belief model [HBM], protection motivation theory [PMT], theory of planned behavior [TPB]) and technology acceptance (unified theory of technology acceptance and use 1 and 2 [UTAUT1 and UTAUT2]) to predict adoption intentions (and frequency) of current COVID-19 contact tracing app use. Relevant variables that are not represented in TPB or UTAUT are italicized.

Third, we aimed to expand upon research on either model by considering the distinct impact of injunctive and descriptive social norms, which have often been neglected in previous research, for instance, regarding contact tracing app use (eg, [25,26]). Thus, we provide an integrative framework to test attitudinal predictors of intention and behavior concerning contact tracing app use in the general population (Figure 3).

## Methods

### Recruitment

Via a cross-sectional online survey, we explored app use and associated attitudes in a convenience sample of the German population. Participants were recruited via social media (Facebook groups, coronavirus-related websites, YouTube), press outlets (local news report, Press and Media Relations Office of the University of Greifswald), and personal communications. The survey was pretested via cognitive debriefings and pilot surveys in a small sample (n=20) for clarity, readability, accessibility, and functioning. During the pretest, we observed a duration of 10-60 minutes for survey completion (depending on literacy, familiarity with surveys, etc), which we established as a guideline for data collection. The survey was subsequently implemented using SoSci Survey (SoSci Survey GmbH) [58], over a period of 3 months (May to July 2020). This recruitment period was chosen to capture a similar time frame of about 4 weeks before and after the official launch of the German Corona Warn-App. Amidst the data collection period, the Corona Warn-App was launched by the German Ministry of Health (on June 16), so we coded participation before or after this launch to control for exposure effects.

The Corona Warn-App uses a decentralized approach to infection tracking. Via Bluetooth, it measures the distance between the smartphones of app users and exchanges temporary encrypted random codes across devices. These codes are cryptographically derived several times per hour based on a random device key. If app users test positive for the coronavirus, they can choose to pass on their keys for matching with other app users who have received the random code from this device in the given time frame. If there is a match, an algorithm determines individual risk (eg, based on estimated physical distance, timing, and duration of the meeting), and subsequently informs app users about their risk status (low or increased risk). The app is available in the App Store and the Google Play Store, and it works with multiple operating systems, starting with iOS 13.5 and Android 6.

As an incentive, 50 vouchers (€15; US $18.24; exchange rate on May 11, 2021) were randomly distributed among participants. Moreover, after study completion, participants were provided additional information on COVID-19 and tracing apps, including several hyperlinks to freely available tracing apps. The study procedure was approved by the local Ethics Committee (blinded for peer review). A list of the items is presented in Multimedia Appendix 1.

### Measurement Instruments

#### Sociodemographic Data

We measured individual age, gender (1=female, 2=male), number of persons in one’s household, current level of education (0=lower secondary education or less; 1=upper secondary education, ie, “Abitur” or higher educational achievement), current personal income (USD values based on the exchange rate on May 11, 2021; 1=€0-€500 [US $0-$608.23], 2=€501-€1000 [US $609.44-$1216.45], 3=€1001-€1500 [US $1217.67-$1824.68], 4=€1501-€2000 [US $1825.90-$2432.91], 5=€2001-€2500 [US $2434.13-$3041.14], 6=€2501 or more [US$ 3042.35 or more]; dummy coded for the analysis with the first category as a reference category), region (0=rural, ie, up to 10,000 inhabitants; 1=urban, ie, up to 100,000 inhabitants; 2=metropolitan, ie, more than 100,000 inhabitants; dummy coded with rural as a reference category), and migration background (1=father/mother/participant born in Germany, 2=father/mother/participant born elsewhere).

#### Health Behavior Change

Core variables of health behavior change were assessed based on the TPB, the PMT, and the HBM (Figure 1) in line with previous studies and recommendations on scale development (eg, [53,59,60]). Mean scores were used for all scales, with higher values representing more positive attitudes.

To capture subjective norms, descriptive personal norms (eg, most people who are important to me want to use such an app) were assessed via 3 items (Cronbach α=.89) and injunctive personal norms (eg, my family expects me to use such an app) via 4 items (α=.84) on a 5-point scale from 1 (strongly disagree) to 5 (strongly agree). In addition, descriptive social norms (α=.75) (eg, how many people in your age group would like to use such an app?) and injunctive social norms (α=.97) (eg, how many people in your age group should use such an app?) were assessed with 2 items each on a scale from 0% to 100%, recoded into 10 categories (0-10, 11-20, etc) for further analysis. Perceived behavioral control was reflected by its subcomponents self-efficacy (eg, I am confident that I could use such an app) via 4 items (α=.81), and perceived controllability (eg, the decision to use such an app is up to me) via 2 items (α=.69) on a 5-point scale from 1 (strongly disagree) to 5 (strongly agree). Attitudes toward app use were assessed with 4 items (eg, good-bad, helpful–not helpful) on a 7-point semantic differential, recoded to represent positive attitudes toward app use (α=.89).

We tested the factor structure of the TPB model via a principal component analysis with varimax rotation (Multimedia Appendix 2). The resulting model (Kaiser-Meyer-Olkin [KMO]=0.80) explained about 70% of cumulative variance and achieved sufficient differentiation with item loadings above .64 on each factor. The factors largely mirrored the constructs attitudes, subjective norms, and perceived behavioral control; however, items measuring injunctive and descriptive personal norms represented one factor and were thus combined into a scale of personal norms (α=.90). Moreover, the items measuring injunctive social norms (TPB_ISN1, TPB_ISN2; see Multimedia Appendix 1) were strongly associated with the factor labelled attitudes but were kept as a separate scale for theoretical reasons.

#### UTAUT

To capture additional constructs described in UTAUT I, we measured performance expectancies (ie, perceived usefulness) via 6 items (eg, a personalized COVID-19 tracing app improves tracing of infection chains; α=.93) as well as the perceived costs of or barriers to app use (eg, using the app can cause problems with other apps; α=.81) on a 5-point Likert scale.

We also assessed perceived ease of use (eg, learning to use the app would be easy for me) with 4 items (α=.91) on a 5-point scale from 1 (strongly disagree) to 5 (strongly agree). To further capture the constructs specified in UTAUT2, we assessed hedonic motivation (how would you feel if you used a COVID-19 tracing app) via 3 semantic differentials (happy-sad, concerned–not concerned, satisfied-dissatisfied; α=.77), price value by eliciting the perceived material benefits of not using the app with 5 items (eg, it saves time or money; α=.88), inversed to represent positive price value, and habit (eg, using an app is something that I do often) with 6 items (α=.91) on a scale from 1 (strongly disagree) to 5 (strongly agree). All scales for the UTAUT assessment were adapted from a previous study in a German sample [61] and were in line with prior UTAUT research (eg, [48,53]). To capture experience, we also asked participants how many hours per day they spent using smartphone apps (analyzed as integers).

Like the TPB model, we examined the structure of the UTAUT model with a principal component analysis with varimax rotation (Multimedia Appendix 3). The resulting model (KMO=0.90) explained about 66% of the cumulative variance and achieved sufficient differentiation with item loadings above .58 on each factor. The factors mirrored the constructs perceived barriers, perceived ease of use, price value, and habit. However, items measuring hedonic motivation were associated with perceived usefulness but were kept as a separate scale for theoretical reasons.

As potential barriers to app use, we also included data privacy concerns (eg, a good privacy policy for mobile app users should have a clear and conspicuous disclosure) measured by the App Information Privacy Scale [62], which consists of 17 items (α=.91) rated on a 7-point Likert scale, and personalization of the app (eg, the app provides information that is exactly tailored to my needs) via 3 items (α=.90), rated on a 5-point Likert scale, to complement the analysis model.

#### COVID-19–Related Information

We included COVID-19–related information on risk perception, namely perceived personal susceptibility to a COVID-19 infection, as well as susceptibility of one’s social surroundings and one’s age group (α=.88), on a scale from 0% to 100%. Values were recoded into 10 categories (0-10, 11-20, etc) for further analysis, similar to social norms. We also measured anticipatory anxiety regarding an infection (oneself, close friends/family; α=.83) on a 5-point scale from 1 (not at all worried) to 5 (very worried), and anticipated emotion as a proxy for emotional severity in case of an infection (eg, sadness, anxiety) via 5 items (α=.81) on a 5-point Likert scale. We assessed subjective knowledge about COVID-19 tracing apps (on a scale of 0 to 100, recoded from 1 to 10), and direct or indirect experience (ie, a close friend or family) with COVID-19, including physical (eg, respiratory illness), material (eg, unemployment), and psychological (eg, depression) consequences (0=no, 1=yes).

Finally, we assessed individual intentions to use a COVID-19 tracing app within the next 3 months via 3 items on a scale of 1 (highly unlikely) to 7 (highly likely) (eg, I plan to use a tracing app within the next 3 months; α=.99). Due to the high correlation between items, they were collapsed into a single indicator with the maximum value across all three representing individual intentions. We also measured the frequency of current tracing app use from 1 (never) to 6 (multiple times per day). To compare app users to nonusers, frequency was dichotomized into 0 (nonusers) and 1 (users) for descriptive analysis. Because the survey was implemented before the official launch of the German Corona Warn-App, response options comprised a variety of available tracing apps (eg, ito App, CoroNotes, Datenspende App) and an open-ended category (other). For analysis purposes, however, all data were collapsed to reflect (the frequency of) tracing app use via single-item measures to estimate use patterns of any kind of tracing app. To make sure that participants responded within a similar frame of reference, we provided a short explanation regarding contact tracing apps before proceeding to ask app-specific questions. This explanation described the functionality of contact tracing apps as (1) monitoring and tracking infection chains, (2) delivering immediate support and information in case of an infection or contact with an infected person, (3) and possibly providing support for persons in quarantine by monitoring health and tailoring information and preventive actions (Multimedia Appendix 4). This definition is in line with current conceptualizations of contact tracing apps [1,3,4]. However, since our study preceded the official launch of the Corona Warn-App, it was impossible to precisely describe this app and its functionalities as a point of reference. We also controlled for exposure to the app via the official launch (0=completed the survey before the official launch, 1=completed the survey after the official launch of the tracing app in Germany on June 16).

### Statistical Analysis

First, we provided a descriptive analysis of the sample (including missing data) and compared current app users to nonusers via *t* tests and chi-square tests. Second, we examined bivariate associations of study variables. For bivariate correlations, we report Pearson and point biserial correlation coefficients, with boundaries of r≥0.1 (weak), r≥0.3 (moderate), and r≥0.5 (strong) as effect sizes. Third, we perform 4 block-wise hierarchical regression models of adoption intentions and frequency of current tracing app use. These models comprise the TPB (model 1), the UTAUT2 (model 2), an integrated model (model 3), and an extended model (model 4) including the additional variables threat appraisals, privacy concerns, and personalization as predictor variables. Because the latter variables are not part of the core set of constructs in either TPB or UTAUT2, we aimed to test their utility beyond the integrated framework using the “additional variables” approach (eg, [48,50]). In each regression model, the first block of predictor variables consisted of covariates, the second block of model components, and the third block of additional variables (if any). By examining the changes in explained variance for each block as well as the regression coefficient of each variable, we can consecutively examine the impact of sociodemographic data, the TPB, the UTAUT2, and additional variables on adoption intentions, and app use. We reported standardized regression coefficients (beta) and R^2^ as effect sizes for each model. All analyses assumed an alpha level of .05 and were conducted with SPSS Statistics 27 (IBM Corp).

## Results

### Descriptive Statistics

In total, 593 persons participated in the survey; however, after excluding participants who completed the entire survey in less than 10 minutes or showed monotone response patterns for >80% of the questions, 349 participants remained (age: mean 35.62, SD 14.66 years; range 18-82 years). On average, participants completed the survey in 22.65 (SD 7.93) minutes. Descriptive statistics are presented in Table 1.

Overall, missing values were low (564/31,759, 1.8%), which the Little test [63] revealed to be missing completely at random (*χ*^2^_2450_=2538.4, *P*=.10). Only 3 variables had more than 5% missing values, namely income (23/349, 6.6%), region (21/349, 6.0%), and an item measuring risk perception (21/349, 6.0%). Therefore, we used complete cases for descriptive statistics and applied pairwise deletion for inferential statistics, as previous studies have shown a low probability of bias in this case [64,65]. In our sample, 19% (67/349) reported current use of a COVID-19 contact tracing app, with an average frequency of several times per week (mean 4.07, SD 1.51). A comparison of current app users and nonusers (*t* tests, chi-square tests) indicates that app use was much higher after the launch and thus exposure to the official Corona Warn-App, and seemingly equally distributed across regions. App users reported significantly more positive attitudes and fewer concerns than nonusers but also had a lower relative frequency of COVID-19 experiences. Perceived controllability of app use did not differ between participants.

**Table 1:** Descriptive statistics comparing current users and nonusers of a COVID-19 contact tracing app.

Characteristic			Total (N=349)	Current app users (n=67)	Nonusers (n=282)	Statistics^a^				
						Value		*P* value		
**Sociodemographic data**										
	Age, mean (SD)		35.62 (14.66)	36.62 (14.73)	35.38 (14.66)	t_333_=0.61		.54		
	Gender (female), n (%)		226 (65.30)	40 (60.60)	186 (67.10)	*χ*^2^_1_=1.0		.31		
	Persons per household, mean (SD)		2.53 (1.58)	2.55 (1.40)	2.54 (1.63)	t_333_=0.11		.92		
	**Education, n (%)**						*χ*^2^_1_=2.8		.10	
		≤Lower secondary	55 (16.50)	15 (23.40)	40 (14.90)					
		Upper secondary	278 (83.50)	49 (76.60)	229 (85.10)					
	**Income, n (%)**						*χ*^2^_5_=6.3		.28	
		€0-€500	66 (20.20)	12 (18.8)	54 (20.6)					
		€501-€1000	70 (21.50)	8 (12.5)	62 (23.7)					
		€1001-€1500	36 (11.00)	9 (14.1)	27 (10.3)					
		€1501-€2000	25 (7.70)	8 (12.5)	17 (6.5)					
		€2001-€2500	46 (14.10)	10 (15.6)	36 (13.7)					
		€2501 or more	83 (25.50)	17 (26.6)	66 (25.2)					
	**Region, n (%)**						*χ*^2^_2_=8.6		.01	
		Rural	66 (20.10)	21 (32.8)	45 (17.0)					
		Urban	143 (43.60)	21 (32.8)	122 (46.2)					
		Metropolitan	119 (36.30)	22 (34.4)	97 (36.7)					
	Migration background^b^, n (%)		76 (21.78)	16 (23.9)	60 (21.5)	*χ*^2^_1_=0.2		.67		
**Theory of planned behavior, mean (SD)**										
	Attitudes (range 1-7)		4.19 (1.65)	4.97 (1.34)	4.01 (1.67)	t_120_=5.00		<.001		
**Subjective norms, mean (SD)**										
	Personal norms (range 1-5)		2.35 (0.87)	2.85 (0.84)	2.23 (0.83)	t_99_=5.43		<.001		
	Injunctive social norms (range 1-10)		5.62 (3.52)	7.69 (2.64)	5.12 (3.53)	t_130_=6.67		<.001		
	Descriptive social norms (range 1-10)		4.62 (1.92)	5.19 (1.89)	4.48 (1.90)	t_101_=2.76		.007		
**Perceived behavioral control, mean (SD)**										
	Self-efficacy (range 1-5)		4.19 (0.85)	4.48 (0.72)	4.12 (0.87)	t_342_=3.12		.002		
	Perceived controllability (range 1-5)		4.31 (0.87)	4.25 (0.95)	4.33 (0.85)	t_342_=0.63		.53		
**Unified theory of acceptance and use of technology, mean (SD)**										
	Perceived ease of use (range 1-5)		4.04 (0.91)	4.37 (0.77)	3.95 (0.92)	t_341_=3.45		.001		
	Perceived usefulness (range 1-5)		3.10 (1.08)	3.54 (0.93)	2.99 (1.09)	t_113_=4.19		*<*.001		
	Perceived barriers (range 1-5)		2.53 (0.79)	2.26 (0.75)	2.60 (0.78)	t_338_=3.15		.002		
	Hedonic motivation (range 1-5)		3.80 (1.31)	4.63 (1.18)	3.60 (1.26)	t_337_=6.02		*<*.001		
	Price value (range 1-5)		2.97 (1.08)	3.44 (1.15)	2.86 (1.03)	t_337_=3.95		*<*.001		
	Habit (range 1-5)		3.46 (1.08)	3.82 (0.95)	3.40 (1.07)	t_344_=2.97		.003		
	Experience (ie, hours of app use per day) (range 0-12)		2.63 (1.78)	2.82 (1.34)	2.61 (1.86)	t_122_=1.02		.31		
**Additional variables**										
	**Threat appraisals**									
		Perceived susceptibility (range 1-10), mean (SD)	4.14 (2.18)	4.31 (2.14)	4.10 (2.19)	t_336_=0.68		.50		
		Anticipatory anxiety (range 1-5), mean (SD)	2.95 (1.03)	3.41 (0.94)	2.85 (1.02)	t_337_=4.02		*<*.001		
		Anticipated emotion (range 1-5), mean (SD)	2.52 (0.88)	2.66 (0.91)	2.48 (0.88)	t_337_=1.43		.16		
	**Other variables**									
		Data privacy concerns (range 1-7), mean (SD)	5.71 (0.85)	5.34 (1.08)	5.79 (0.76)	t_344_=4.00		<.001		
		Personalization (range 1-5), mean (SD)	2.88 (1.15)	3.36 (1.14)	2.76 (1.12)	t_344_=3.89		<.001		
		Subjective knowledge about COVID-19 tracing apps (range 1-10), mean (SD)	3.69 (2.36)	5.05 (2.41)	3.37 (2.24)	t_324_=5.27		<.001		
		COVID-19 experience, n (%)	161 (47.60)	23 (35.4)	138 (50.50)	*χ*^2^_1_=4.8		.03		
		Exposure (ie, surveyed after the Corona Warn-App launch), n (%)	135 (38.68)	54 (80.6)	81 (29.0)	*χ*^2^_1_=60.4		<.001		
		Adoption intentions (range 1-7), mean (SD)	3.66 (2.37)	5.78 (1.94)	3.15 (2.17)	t_110_=9.71		<.001		

^a^Two-tailed *t* test or chi-square test results.

^b^Either the participant, their mother, or their father was not born in Germany.

### Bivariate Correlations

Sociodemographic data were not associated with adoption intentions, but frequency of current tracing app use was negatively correlated with education (r=–0.13, *P*=.02) and urban region (r=–0.16, *P*=.005), indicating that fewer educated participants living in metropolitan or rural areas reported more frequent tracing app use (Multimedia Appendix 5).

Regarding TPB and UTAUT constructs, Table 2 shows that adoption intentions were moderately to strongly associated with most TPB constructs (r=0.38, *P*<.001 to r=0.69, *P*<.001), except for controllability (r=0.03, *P*=.63), as well as UTAUT constructs (r=0.29, *P*<.001 to r=0.64, *P*<.001) except for experience (r=0.10, *P*=.09). Perceived costs (r=–0.35, *P*<.001) were negatively linked to intentions. Overall, frequency of current app use showed similar but weaker associations. Exposure to the app was not associated with any attitudinal variable but was associated with intentions (r=0.11, *P*=.04) and frequency of app use (r=0.39, *P*<.001).

Additionally, TPB and UTAUT constructs correlated considerably with attitudes and injunctive social norms (r=0.68, *P*<.001), attitudes and perceived usefulness (r=0.80, *P*<.001), attitudes and hedonic motivation (r=0.71, *P*<.001), with hedonic motivation and perceived usefulness (r=0.69, *P*<.001) being particularly high, underlining their conceptual similarities (cf principal component analyses in Multimedia Appendices 2 and 3). Adoption intentions and use frequency were moderately correlated (r=0.46, *P*<.001).

Among additional variables (Multimedia Appendix 5), adoption intentions were mostly strongly associated with personalization (r=0.57, *P*<.001), followed by anticipatory anxiety (r=0.41, *P*<.001), data privacy concerns (r=–0.30, *P*<.001), and anticipated emotion (r=0.24, *P*<.001). Frequency of current tracing app use had similar associations and was also positively correlated with knowledge about tracing apps (r=0.28, *P*<.001).

**Table 2:** Bivariate correlations between core constructs of health behavior models, technology acceptance, adoption intentions, and frequency of use of COVID-19 contact tracing apps.

Constructs	1	2	3	4	5	6	7	8	9	10	11	12	13	14	15	16
1. Attitudes	1															
2. Personal norms	.51^a^	1														
3. Injunctive social norms	.68^a^	.60^a^	1													
4. Descriptive social norms	.43^a^	.52^a^	.48^a^	1												
5. Self-efficacy	.37^a^	.35^a^	.33^a^	.31^a^	1											
6. Perceived controllability	.08	.06	–.04	.05	.22^a^	1										
7. Perceived usefulness	.80^a^	.48^a^	.66^a^	.41^a^	.36^a^	–.02	1									
8. Perceived barriers	–.35^a^	–.23^a^	–.26^a^	–.22^a^	–.35^a^	–.04	–.28^a^	1								
9. Perceived ease of use	.29^a^	.30^a^	.29^a^	.27^b^	.64^a^	.07	.25^a^	–.26^a^	1							
10. Hedonic motivation	.71^a^	.48^a^	.60^a^	.39^a^	.31^a^	.03	.69^a^	–.33^a^	.26^a^	1						
11. Price value	.41^a^	.26^a^	.34^a^	.26^a^	.25^a^	–.07	.36^a^	–.52^a^	.19^a^	.36^a^	1					
12. Habit	.35^a^	.19^a^	.27^a^	.23^a^	.40^a^	.04	.32^a^	–.23^a^	.37^a^	.32^a^	.25^a^	1				
13. Experience	.06	.02	.06	.14^c^	.12^c^	.02	.07	–.05	.20^b^	.10	.11	.42^a^	1			
14. Adoption intentions	.66^a^	.63^a^	.69^a^	.48^a^	.38^a^	.03	.63^a^	–.35^a^	.31^a^	.64^a^	.42^a^	.29^a^	.10	1		
15. App use frequency	.21^b^	.32^a^	.27^a^	.15^b^	.15^b^	.04	.12^c^	–.17^b^	.20^a^	.28^a^	.15^b^	.14^b^	.06	.46^a^	1	
16. Exposure	.02	.05	.05	.03	.01	.07	.00	–.04	.02	–.03	.07	–.04	–.06	.11^c^	.39^a^	1

^a^*P*<.001.

^b^*P*<.01.

^c^*P*<.05.

### Hierarchical Regression Models

Results of regression models testing the TPB (model 1), the UTAUT (model 2), the integrated model (model 3), and the extended model (model 4) are presented in Table 3. In each analysis, we included several covariates in the first block (ie, age, gender, and education; number of persons per household, income, and region; as well as subjective knowledge regarding COVID-19 tracing apps, COVID-19 experience, and exposure). The selection of covariates was based on previous research on technology use, and preventive behaviors during the COVID-19 pandemic. The second block contained TPB (model 1) or UTAUT constructs (model 2) or both (model 3). The third block contained additional variables (model 4). Due to high correlations between some variables, we calculated the variance inflation factor (VIF) to test for multicollinearity [66]. Accordingly, a VIF of more than 4 is often considered an indicator of multicollinearity. The VIF was below 3.00 in model 1 and model 2, but in model 3 and 4, the VIF ranged from 1.11 (COVID-19 experience) to 4.04 (attitudes). Due to its high VIF, high bivariate correlations with other constructs, and its conceptual similarities to perceived usefulness, we excluded the variable measuring attitudes from models 3 and 4.

The TPB (model 1) explained 63% of adoption intentions, with injunctive social norms (β=.35, *P*<.001), attitudes (β=.28, *P*<.001), and personal norms having the strongest association (β=.22, *P*<.001); self-efficacy having a smaller effect (β=.08, *P*=.04); and perceived controllability being not significant (β=–.02, *P*=.56). Regarding current app use (R^2^=0.33), personal norms (β=.20, *P*=.004) were connected to more frequent use, but exposure to the warning app had the strongest association (β=.31, *P*<.001). Additionally, persons from more densely populated areas reported less frequent app use.

The UTAUT (model 2) explained 56% of adoption intentions and 37% of frequent app use. Perceived usefulness (β=.32, *P*<.001) and hedonic motivation (β=.35, *P*<.001) were among the strongest predictor variables of intentions and frequency of use; however, the influence of perceived usefulness was inverted (β=–.14, *P*=.049) for frequency of use. Perceived ease of use, perceived barriers, and experience were not significant. Price value was positively associated with intentions (β=.13, *P*=.02) but not frequency, while habit was associated with frequency (β=.15, *P*=.02) but not intentions.

The integrated model (model 3) explained 67% of intentions with injunctive social norms (β=.31, *P*<.001), hedonic motivation (β=.22, *P*<.001), personal norms (β=.21, *P*<.001), and price value (β=.11, *P*=.02) remaining significant. Regarding frequency, the model explained 40% of the variance with personal norms (β=.18, *P*=.007), hedonic motivation (β=.34, *P*<.001), habit (β=.14, *P*=.03), and perceived usefulness (β=–.23, *P*=.004).

Finally, the extended model with the additional constructs privacy concerns, personalization, and threat appraisals (model 4) performed marginally better regarding intentions (R^2^=0.68) and frequency of app use (R^2^=0.42). Anticipatory anxiety was also significantly associated with intention (β=.09, *P*.04) and frequency (β=.15, *P*=.03). In addition, privacy concerns were associated with less frequent app use (β=–.09, *P*=.046).

**Figure 4:**
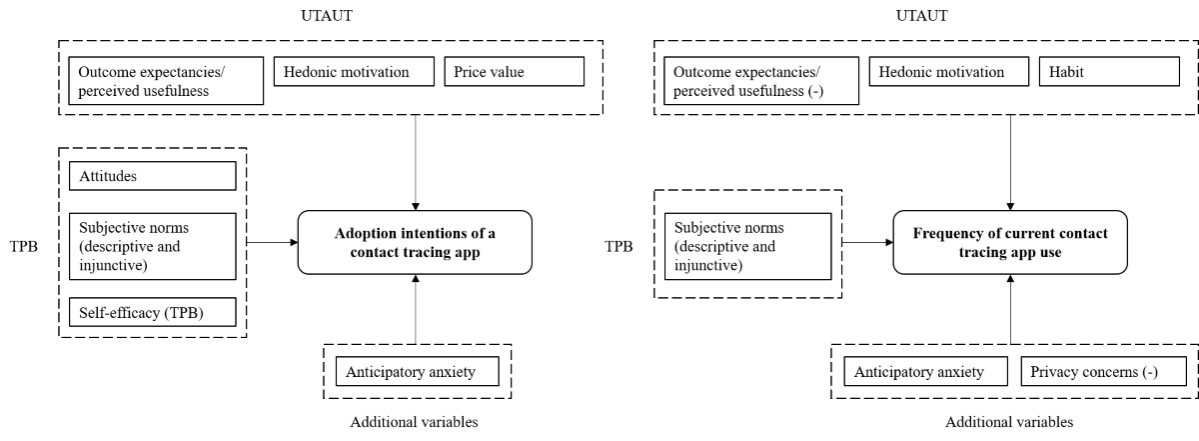
Significant predictor variables of adoption intentions and (frequency) of current COVID-19 contact tracing app use in a German community sample. Predictors are categorized according to the theory of planned behavior (TPB), the unified theory of technology acceptance and use (UTAUT), and additional variables from other health behavior models (ie, anticipatory anxiety) or previously extended UTAUT models (ie, data privacy concerns).

To summarize, the analysis showed that the TPB as well as the UTAUT proved useful in determining intentions, and frequency of use of a COVID-19 contact tracing app, although model configurations varied greatly between both outcomes (Figure 4). While most of the constructs of the TPB were significantly associated with intentions (except for controllability), neither perceived barriers, ease of use, nor experience reached significance within the UTAUT framework. Overall, perceived usefulness, subjective norms, hedonic motivation, and anticipatory anxiety were consistently associated with tracing app use across models.

**Table 3:** Standardized regression weights and explained variance of hierarchical regression models predicting adoption intentions and frequency of current contact tracing app use.

Variable				TPB^a^ (model 1)					UTAUT^b^ (model 2)					Integrated model (model 3)					Extended model (model 4)		
				Intention		Frequency		Intention			Frequency		Intention			Frequency		Intention			Frequency
**Block 1**																					
	**Covariates**																				
		Age	.02		.03		.12			.14		.05			.10		.06			.12	
		Gender (ref^c^: female)	–.02		–.01		.05			.00		.00			–.03		.01			–.02	
		Migration background	.03		.01		.04			.01		.04			.02		.04			.03	
		Education (ref: ≤lower secondary)	–.02		–.10		–.02			–.08		–.02			–.07		–.02			–.08	
		Persons per household	–.01		–.01		–.02			.01		–.02			–.01		–.02			–.01	
	**Income (ref: €0-€500)**																				
		€501-€1000	.02		.02		.00			–.01		–.01			–.01		.00			.00	
		€1001-€1500	.03		.03		–.01			.02		.01			.02		.01			.02	
		€1501-€2000	–.05		–.03		–.09			–.07		–.10^d^			–.06		–.09			–.05	
		€2001-€2500	.05		–.01		–.03			–.05		.01			–.04		.02			–.03	
		€2501 or more	.06		.05		–.04			–.04		.02			–.02		.03			–.02	
	**Region (ref: rural)**																				
		Urban	–.06		–.18^d^		–.04			–.17^d^		–.04			–.18^e^		–.05			–.18^e^	
		Metropolitan	–.01		–.16^d^		–.01			–.14^d^		.01			–.14^d^		.00			–.14^d^	
		Subjective knowledge^f^	.06		.17^g^		.09^d^			.21^g^		.07			.20^g^		.05			.18^e^	
		COVID-19 experience	.01		–.05		–.02			–.08		–.01			–.07		.00			–.06	
		Exposure^h^	.05		.31^g^		.08			.34^g^		.06			.33^g^		.06			.33^g^	
**Block 2**																					
	**TPB**																				
		Attitudes	.28^g^		.05		—^i^			—		—			—		—			—	
		Personal norms	.22^g^		.20^e^							.21^g^			.18^e^		.19^g^			.16^d^	
		Injunctive social norms	.35^g^		.13		—			—		.31^g^			.11		.29^g^			.08	
		Descriptive social norms	.05		–.07		—			—		.03			–.08		.04			–.07	
		Self-efficacy	.08^d^		.07		—			—		.03			.01		.04			.02	
		Controllability	–.02		–.01		—			—		.02			–.02		.02			–.02	
	**UTAUT**																				
		Perceived usefulness	—		—		.32^g^			–.14^d^		.11			–.23^e^		.05			–.25^e^	
		Perceived barriers	—		—		–.04			–.02		–.03			–.03		–.02			–.02	
		Perceived ease of use	—		—		.08			.08		–.01			.05		–.03			.04	
		Habit	—		—		.07			.15^d^		.04			.14^d^		.03			.12	
		Price value	—		—		.13^d^			–.05		.11^d^			–.06		.11^d^			–.05	
		Hedonic motivation	—		—		.35^g^			.39^g^		.22^g^			.34^g^		.21^g^			.32^g^	
		Experience	—		—		.00			–.01		.03			.02		.02			.01	
**Block 3**																					
	**Additional variables**																				
		Perceived susceptibility	—		—		—			—		—			—		–.08			–.05	
		Anticipatory anxiety	—		—		—			—		—			—		.09^d^			.15^d^	
		Anticipated emotion	—		—		—			—		—			—		.01			–.04	
		Data privacy concerns	—		—		—			—		—			—		–.04			–.09^d^	
		Personalization	—		—		—			—		—			—		.09			.00	
**Correlations (R^2^)**																					
	Block 1			.04		.23		.04			.23		.04			.23		.04			.23
	Block 2			.63		.33		.56			.37		.67			.40		.67			.40
	Block 3			—		—		—			—		—			—		.68			.42

^a^TPB: theory of planned behavior.

^b^UTAUT: unified theory of acceptance and use of technology.

^c^ref: reference.

^d^*P*<.05.

^e^*P*<.01.

^f^Subjective knowledge about COVID-19 tracing apps.

^g^*P*<.001.

^h^Exposure (ie, surveyed after Corona Warn-App launch).

^i^Not applicable.

## Discussion

### Principal Results

This study aimed to investigate the utility of health behavior theories and technology acceptance models for explaining adoption intentions and current use of a contact tracing app during the COVID-19 pandemic in the German population. The TPB as well as the UTAUT explained between 56% (UTAUT) and 63% (TPB) of adoption intentions as well as 33% (TPB) and 37% (UTAUT) of current app use. Extended models including threat appraisal, privacy concerns, and personalization features of the app explained an additional 5% for both outcomes. Overall, exposure to the app (ie, study participation following an official app launch with governmental support) was strongly associated with frequent app use and was also associated with greater acceptance and more positive attitudes (Table 1). In accordance with previous research, both models have greater predictive value concerning intentions than behavior [40], which might be connected to the intention-behavior gap [38] and related factors, such as a lack of familiarity with the app, and the unprecedented and ever-changing nature of the pandemic.

### Comparison With Prior Work

A closer look at the regression models also reveals that while the main components of the TPB were significantly associated with intentions—except for controllability—this was not the case for the UTAUT model. In fact, only perceived usefulness and hedonic motivation were highly significant and consistent, while perceived barriers, experience, and ease of use were not significant. Habit and price value played a significant role for some but not all associations. Nevertheless, the large amount of explained variance points to the model’s utility in explaining adoption intentions. In general, our findings support previous research on COVID-19 contact tracing apps, as we also observed strong positive associations between response efficacy (ie, perceived usefulness in UTAUT terms) and adoption intentions, but only moderate positive associations for self-efficacy [25-27]. Presumably, high ratings of perceived usefulness or positive attitudes toward the app (ie, the app being perceived as helpful; Multimedia Appendix 1) are also associated with trust in flawless performance, a low rate of false positives, and thus a sense of security. While we did not assess these constructs directly, we assume this association based on previous research on human-computer interaction [8] as well as warning systems [9]. However, future research in this context could examine the associations between trust, perceived security, and positive attitudes (eg, perceived usefulness) more closely, for instance, by using factorial surveys that vary attributes of tracing apps (eg, rate of false alarms, sensitivity) and subsequently measure attitudes [21,67].

Interestingly, the association of perceived usefulness and frequent app use was inversed, meaning that higher perceived useful was associated with lower use frequency. Given that tracing apps are designed to inform about potential COVID-19 infections and thus serious health risks, high efficacy beliefs (or perceived usefulness) would mean that more frequent app use might increase the chance of receiving health warnings which could be perceived as a negative and undesirable event. Thus, using the app less frequent could be seen as avoiding a potential threat by reducing the information flow. This risk avoidance behavior is a common phenomenon in risk research, for example, within the risk perception attitude framework [68] that connects information avoidance to high risk perceptions as well as lower self-efficacy and control beliefs. On the other hand, if perceived usefulness is low, potential health warnings issued by the app might be taken less seriously due to a higher possibility of false positives. Since only a very frequent app use guarantees preventive efficacy of the tracing app [1,3,4], these findings are concerning. We believe that future mixed methods research can elucidate the role of perceived usefulness as a potential barrier to frequent app use as well as its interaction with other predictors, such as self-efficacy and risk perception.

Furthermore, we also observed consistent significant associations between anticipatory anxiety and (frequent) app use, although the effect was small. Conceptually, these appraisals can reflect health concerns [35,60], which are associated with vigilance toward symptoms or health problems. Since a tracing app provides an additional source of information regarding a potential illness, it can help to alleviate concerns and support vigilance [1]. Similarly, frequent app use could also increase one’s sense of security and positive feelings, which is mirrored by a positive association with hedonic motivation (eg, feeling satisfied, rather happy than sad, and less concerned after using the app). A similar trade-off between mHealth use, user concerns, and perceived security has already been established (eg, [1,69]). This hypothesized association between hedonic motivation, sense of security, and technology use should therefore be tested in longitudinal studies on tracing app use.

While our study underlines the importance of attitudes and perceived usefulness, we also found a very strong connection between social norms, namely personal norms (ie, referring to one’s immediate surroundings), intentions, and behavior, which is contrary to prior research on tracing app use [27]. However, as previously mentioned, Kukuk [27] compiled a measure of social influence [45] that blended subjective norms, social support, and instrumental governmental support, which might be confounded by other factors such as trust in the government. Evidently, Kukuk’s measure was insufficient in capturing social norms as it showed very low factorial and content validity. Additionally, in this study, injunctive social norms (ie, referring to perceived societal expectations) were also strongly connected to adoption intentions across all models. The strong impact of social norms on behavioral prevention has already been documented for previous pandemics [28,29]. With infection prevention measures (eg, social distancing) affecting the heart of social interaction, it seems reasonable to also assume a strong impact of social norms. If one’s close social network is believed to support the app, one is more likely to also engage with it, leading to a social group that protects its members by using the app because of the accelerated communication in case of an infection [5]. Thus, the (frequent) use of a tracing app can be seen as an act of social responsibility. This has implications for health communication. Promoting app use as an expression of social responsibility and putting less emphasis on the perceived usefulness might be helpful in increasing public acceptance avoid potential side effects (cf the role of perceived usefulness in this study). In this sense, the approach would be quite similar to successful nudging interventions used to foster technology use [55] or vaccine uptake [56] that are based on social norms.

Moreover, two additional components of the UTAUT were significantly associated with intentions (price value) and frequency of app use (habit). In this study, price value was not solely defined by monetary restrictions [51] because the German contact tracing apps are available free of charge. Hence, we also included other limited resources (eg, time) as a potential restriction or cost. In general, the positive association with adoption intentions was in line with previous research [50,51]; however, since our operationalization of price value also included time, it is possible that the effect disappeared for frequency of use because of a foreseeable increase in time investment. Therefore, we hypothesize a trade-off between perceived costs (ie, time investment) and benefits that might differentially affect intentions and behavior. This would also explain the observed association of habit with frequency of use but not general adoption intentions. Habit describes prior experience with a technology but also the belief that using said technology is automatic [51]. Automatic behaviors are associated with less effort because it is no longer necessary to carefully and consciously plan and monitor them (eg, [53]). Since less effort equals less time spent on (consciously) using the app, habitual use may buffer the costs of more frequent app use (ie, a greater time investment). A similar trade-off is described for effort expectancies and performance expectancies regarding technologies [50] and health behavior models (eg, [25,60]) in that a positive balance of perceived benefits over efforts predicts intentions and behaviors. However, while effort expectancies often focus on singular behaviors instead of resources, it might be beneficial to weigh perceived resource investment and behavioral efforts when predicting intentions in future studies.

Finally, we also addressed some ethical challenges that might arise in this context, namely the age-related digital divide [10,12] and privacy concerns (eg, [1,6,7]). In contrast to previous studies, we did not find any direct link between age and intentions or behavior, but older age was positively associated with privacy concerns and negatively with personalization (Multimedia Appendix 5). The negative association with personalization might reflect a lack of competence in app use in older participants, as personalizing apps requires certain skills [11,12]. Likewise, increased privacy concerns might also be associated with increased insecurity and a lack of knowledge about procedures of privacy protection implemented in the app. To concede, this study was an online study and therefore potentially excluded people who are less interested or competent in using digital technologies. However, the possibility of an indirect negative effect of age on tracing app use via privacy concerns and lower perceived benefits (ie, personalization) is alarming and has implications for health communication research. Interventional studies are needed to test whether tailored health communication surrounding benefits and privacy concerns can alleviate concerns and lead to higher acceptance and adoption of tracing apps in older participants.

Privacy concerns were negatively associated with app use, which underscores the importance of a transparent, sound, and secure data management plan when developing and promoting mHealth apps [14]. Although the effect was rather small, it is possible that participants do indeed fear misuse of health information beyond COVID-19 purposes, providing further empirical support for current publications on the promises and pitfalls of digital health solutions during the pandemic (eg, [3,6,7]). Nevertheless, further research is necessary to explore privacy concerns and their role in determining nonuse of contact tracing apps and other digital health solutions during the COVID-19 pandemic.

### Limitations

The study has several limitations. First, the sample is a German convenience sample that is not representative of the population. The survey was conducted as a self-administered online survey; the reliance on self-reports might have affected our results; hence, future studies should include objective measures (eg, smartphone data on app use). In addition, although we checked the responses for monotone response patterns, we did not incorporate attention checks into the online survey. Second, regarding the cultural context of our study, we acknowledge that the technical, legal, and cultural conditions of contact tracing apps and their implementation varies greatly across countries worldwide. Since we started our survey before the official launch of the Corona Warn-App in Germany, our results are not tailored to this app but rather tracing apps in general, which might explain why we found generally lower associations for current use than for use intentions. Hence, the findings should be replicated in larger, international samples and ideally cross-cultural studies. Third, the study is cross-sectional in nature, and it was therefore not possible to longitudinally link attitudes, intentions, and behaviors as intended in the theoretical models. While strong positive exposure effects were observed, this could also point to selection bias, where proponents of the app were more strongly motivated to participate in the survey than opponents. Fourth, the measures should be tested regarding their psychometric properties. Although the instruments were based on recommendations for behavior change research, and adapted from previous research projects, their implementation in this novel context proved challenging in some areas and requires further investigation. Fourth, other constructs like trust in the government, the app provider, or health communication about COVID-19 should be included to test their impact on attitudes and the path toward adoption of the app. For example, research on the UTAUT model (eg, [48,50]) has coined the “additional variables” approach, where factors are added to the traditional UTAUT model to inspect their incremental validity. Despite these limitations, the study was based on established theories of health behavior change and technology acceptance and implemented in an ecologically valid setting. Further, it provides some insight into urgent processes of health communication and infection prevention that can support the fight against the global COVID-19 pandemic by increasing the use of contact tracing apps in the general population.

### Conclusions

This study examined the utility of health behavior change and theory acceptance models in exploring intentions and use of a contact tracing app to complement preventive measures during the COVID-19 pandemic. In general, both models provide useful information for identifying core beliefs that affect intention and behavior. Among them, subjective norms, hedonic motivation, perceived usefulness, and anticipatory anxiety were particularly important across all tested models. Thus, it seems that promulgating positive social norms and addressing health concerns in health communication might be particularly beneficial to increase tracing app use in the population. The role of perceived usefulness and tracing app use needs further investigation due to its inverse associations with intentions and behavior. Moreover, privacy concerns also emerged as a barrier to app use in this context, underlining the need for more transparency and education regarding data security in the general population. Overall, the official launch of the Corona Warn-App [23] with governmental support seemed to boost awareness and app use in the population; therefore, a concerted effort is recommended when introducing a contact tracing app as an official complementary measure of infection prevention. However, data were collected between May and July 2020 amidst the first wave of the COVID-19 pandemic, so future research needs to illustrate how living under pandemic circumstances affects acceptance and use of (preventive) technology in the long term.

## Appendix

Multimedia Appendix 1 List of items (translated from German to English).

Multimedia Appendix 2 Factor loadings (above .30) of the rotated matrix of items representing the theory of planned behavior.

Multimedia Appendix 3 Factor loadings (above .30) of the rotated matrix of items representing the unified theory of acceptance and use of technology.

Multimedia Appendix 4 Description of a COVID-19 contact tracing app.

Multimedia Appendix 5 Bivariate correlations (Pearson, point biserial) between sociodemographic data, threat appraisals, additional attitudinal variables (personalization, data privacy concerns), and adoption intentions as well as the frequency of use of COVID-19 contact tracing apps.

## Abbreviations

HBM: health belief model
KMO: Kaiser-Meyer-Olkin
mHealth: mobile health
PMT: protection motivation theory
TPB: theory of planned behavior
UTAUT: unified theory of acceptance and use of technology
VIF: variance inflation factor
