# Supplementary material for: Utilizing Health Behavior Change and Technology Acceptance Models to Predict the Adoption of COVID-19 Contact Tracing Apps: Cross-sectional Survey Study
Source: J Med Internet Res. 2021 May 19;23(5):e25447. doi: 10.2196/25447 (PMC8136409; doi:10.2196/25447)
Supplement: Multimedia Appendix 1 [file jmir_v23i5e25447_app1.doc]

**Multimedia Appendix 1**

Supplementary Table S1. List of items (translated from German to English)

| Construct | Item | Text |
| --- | --- | --- |
|  |  |  |
| Age | SD1 | Please state your age (in years). |
| Gender | SD2 | Please indicate the gender you most identify with. |
| Number of persons  in one’s household | SD3 | How many people currently live in your household (including yourself)? |
| Level of education | SD4 | What is your highest academic achievement? |
| Current income | SD5 | Please state your current personal net income. |
| Region | SD6 | Please state the estimated number of inhabitants of the place you currently reside in. |
| Migration background | SD7, SD8, SD9 | In which country were you (SD03)/was your mother (SD04)/was your father (SD05) born? |
| **Attitudes** |  | *How do you rate the use of a contact tracing app?* |
|  | TPB_ATT1 | Useless – useful |
|  | TPB_ATT2 | Harmful – harmless |
|  | TPB_ATT3 | Good – bad |
|  | TPB_ATT4 | Not helpful - helpful |
| **Subjective norms  (injunctive personal norms)** |  |  |
|  | TPB_IPN1 | Most people who are important to me think that I should use such an app. |
|  | TPB_IPN2 | Many people close to me think that I should use such an app. |
|  | TPB_IPN3 | Other people expect me to use such an app. |
|  | TPB_IPN4 | My family expects me to use such an app. |
| **Subjective norms  (Injunctive social norms)** |  |  |
|  | TPB_ISN1 | How many people in your age group should use such an app? |
|  | TPB_ISN2 | How many people in Germany should use such an app? |
| **Subjective norms  (Descriptive personal norms)** |  |  |
|  | TPB_DPN1 | Most people who are important to me want to use such an app. |
|  | TPB_DPN2 | Many people close to me want to use such an app. |
|  | TPB_DPN3 | My family wants to use such an app. |
| **Subjective norms  (Descriptive social norms)** |  |  |
|  | TPB_DSN1 | In your opinion, how many people in Germany want to use such an app? |
|  | TPB_DSN2 | In your opinion, how many people in your age group want to use such an app? |
| **Perceived behavioral control  (self-efficacy)** |  |  |
|  | TPB_SE1 | I am confident that I could use such an app. |
|  | TPB_SE2 | I am sure that I could use such an app. |
|  | TPB_SE3 | I know how to use such an app. |
|  | TPB_SE4 | I am confident in my ability to use such an app. |
| **Perceived behavioral control (controllability)** |  |  |
|  | TPB_C1 | It is up to me to use such an app. |
|  | TPB_C2 | The decision to use such an app is up to me. |
| **Perceived usefulness** |  | *A COVID-19 tracing app …* |
|  | U_PU1 | … improves the tracing of infection chains. |
|  | U_PU2 | … is a good opportunity to reduce my personal infection risk. |
|  | U_PU3 | … improves the implementation and support of quarantine. |
|  | U_PU4 | … reduces the infection risk of other people. |
|  | U_PU5 | … is effective in informing the public about potential infections. |
|  | U_PU6 | … is effective in protecting me against potential infections |
| **Perceived barriers/costs** |  |  |
|  | U_PB1 | Development and implementation of a COVID-19 tracing app is very expensive. |
|  | U_PB2 | Using mobile data for a COVID-19 tracing app is very expensive. |
|  | U_PB3 | It takes a lot of time to use a COVID-19 tracing app (e.g., report personal data). |
|  | U_PB4 | It takes a lot of time to keep a COVID-19 tracing app up and running. |
|  | U_PB5 | Using the app can cause problems with other apps. |
|  | U_PB6 | Using the app takes a lot of time and other resources. |
| **Perceived ease of use** |  |  |
|  | U_PEU1 | My interaction with the app would be clear and easy to comprehend. |
|  | U_PEU2 | It would be easy for me to become competent in using the app. |
|  | U_PEU3 | Using the app would be easy for me. |
|  | U_PEU4 | Learning to use the app would be easy for me. |
| **Hedonic motivation** |  | *How would you feel if you used a COVID-19 tracing app?* |
|  | U_HED1 | Happy – sad |
|  | U_HED2 | Concerned – not concerned |
|  | U_HED3 | Satisfied – dissatisfied |
| **Price value** |  | *Not using a COVID-19 tracing app …* |
|  | U_PV1 | … would save time. |
|  | U_PV2 | … would save money. |
|  | U_PV3 | … would save effort/trouble. |
|  | U_PV4 | … would avoid unnecessary actions. |
| **Habit** |  | *Using an app …* |
|  | U_HAB1 | … is something that I do automatically. |
|  | U_HAB2 | … is something I begin to do before I notice it. |
|  | U_HAB3 | … is something that would be hard for me not to do. |
|  | U_HAB4 | … that I do often. |
|  | U_HAB5 | … that is typical of me. |
|  | U_HAB6 | … that I have been doing for a long time. |
| Experience | U_EXP | How many hours per day do you use mobile applications (e.g., WhatsApp)? |
| **Perceived susceptibility** |  |  |
|  | TA _PS1 | How likely will you contract COVID-19? |
|  | TA _PS2 | How likely will a person close to you (e.g., from your family) contract COVID-19? |
|  | TA _PS3 | How likely will a person of your age group in Germany contract COVID-19? |
| **Anticipatory anxiety** |  |  |
|  | TA_AA1 | How anxious are you that you might contract COVID-19? |
|  | TA_AA2 | How anxious are you that a person close to you (e.g., from your family) might contract COVID-19? |
| **Anticipated emotion** |  | *How would you feel if you contracted COVID-19?* |
|  | TA_AE1 | I would be afraid. |
|  | TA_AE2 | I would be angry. |
|  | TA_AE3 | I would be sad. |
|  | TA_AE4 | I would be hopeless. |
|  | TA_AE5 | I would be depressed. |
| **Data privacy concerns** |  |  |
|  | DP1 to DP17 | *See Buck, C., & Burster, S. (2017). App information privacy concerns.* [The German version was provided via personal communication from the authors] |
| **Personalization** |  |  |
|  | PERS1 | The app provides information that is exactly tailored to my needs. |
|  | PERS2 | The app provides the kind of information that I need. |
|  | PERS3 | The app provides information that is important to the current situation. |
| Subjective knowledge about  COVID-19 contact tracing apps | SK | Please rate your current knowledge about COVID-19 contact tracing apps on a scale from 0 to 100 where 0 indicates no knowledge at all and 100 indicates that you know everything there is to know about contact tracing apps. |
| COVID-19 experience | CE | Have you or a person close to you (i.e. a close friend or family) been affected by COVID-19? |
| **Intention of app use** |  |  |
|  | INT1 | I intend to use a COVID-19 contact tracing app within the next three months. |
|  | INT2 | I plan to use a COVID-19 contact tracing app within the next three months. |
|  | INT3 | Within the next three months, I will very likely use a COVID-19 contact tracing app. |
| Frequency of app use | FREQ | How often do you currently use one or more of the following COVID-19 contact tracing apps? |
