# Supplementary material for: Utilizing Health Behavior Change and Technology Acceptance Models to Predict the Adoption of COVID-19 Contact Tracing Apps: Cross-sectional Survey Study
Source: J Med Internet Res. 2021 May 19;23(5):e25447. doi: 10.2196/25447 (PMC8136409; doi:10.2196/25447)
Supplement: Multimedia Appendix 2 [file jmir_v23i5e25447_app2.doc]

**Multimedia Appendix 2**

Supplementary Table S2. Factor loadings (above .3) of the rotated matrix of items representing the theory of planned behavior (TPB)

|  | Factor 1 | Factor 2 | Factor 3 | Factor 4 | Factor 5 |
| --- | --- | --- | --- | --- | --- |
| **Attitudes** |  |  |  |  |  |
| TPB_ATT1 | .821 |  |  |  |  |
| TPB_ATT2 | .730 |  |  |  |  |
| TPB_ATT3 | .801 |  |  |  |  |
| TPB_ATT4 | .868 |  |  |  |  |
| **Subjective norms** |  |  |  |  |  |
| **Injunctive personal norms** |  |  |  |  |  |
| TPB_IPN1 | .415 | .641 |  |  |  |
| TPB_IPN2 | .300 | .710 |  |  |  |
| TPB_IPN3 |  | .777 |  |  |  |
| TPB_IPN4 |  | .795 |  |  |  |
| **Injunctive social norms** |  |  |  |  |  |
| TPB_ISN1 | .712 | .358 |  |  |  |
| TPB_ISN2 | .733 | .341 |  |  |  |
| **Descriptive personal norms** |  |  |  |  |  |
| TPB_DPN1 | .341 | .694 |  |  |  |
| TPB_DPN2 |  | .706 | .313 |  |  |
| TPB_DPN3 |  | .676 |  |  |  |
| **Descriptive social norms** |  |  |  |  |  |
| TPB_DSN1 |  |  | .864 |  |  |
| TPB_DSN2 | .332 |  | .735 |  |  |
| **Perceived behavioral control** |  |  |  |  |  |
| **Self-efficacy** |  |  |  |  |  |
| TPB_SE1 |  |  |  | .752 |  |
| TPB_SE2 |  |  |  | .704 |  |
| TPB_SE3 |  |  |  | .777 |  |
| TPB_SE4 |  |  |  | .826 |  |
| **Controllability** |  |  |  |  |  |
| TPB_C1 |  |  |  |  | .833 |
| TPB_C2 |  |  |  |  | .861 |
