# Supplementary material for: Utilizing Health Behavior Change and Technology Acceptance Models to Predict the Adoption of COVID-19 Contact Tracing Apps: Cross-sectional Survey Study
Source: J Med Internet Res. 2021 May 19;23(5):e25447. doi: 10.2196/25447 (PMC8136409; doi:10.2196/25447)
Supplement: Multimedia Appendix 3 [file jmir_v23i5e25447_app3.doc]

**Multimedia Appendix 3**

Supplementary Table S3. Factor loadings (above .3) of the rotated matrix of items representing the unified theory of acceptance and use of technology (UTAUT)

|  | Factor 1 | Factor 2 | Factor 3 | Factor 4 | Factor 5 |
| --- | --- | --- | --- | --- | --- |
| **Perceived usefulness** |  |  |  |  |  |
| U_PU1 | .827 |  |  |  |  |
| U_PU2 | .843 |  |  |  |  |
| U_PU3 | .761 |  |  |  |  |
| U_PU4 | .788 |  |  |  |  |
| U_PU5 | .810 |  |  |  |  |
| U_PU6 | .843 |  |  |  |  |
| **Perceived barriers/costs** |  |  |  |  |  |
| U_PB1 |  | .635 |  |  |  |
| U_PB2 |  | .714 |  |  |  |
| U_PB3 |  | .729 |  |  |  |
| U_PB4 |  | .595 |  |  |  |
| U_PB5 |  | .702 |  |  |  |
| U_PB6 | -.307 | .684 |  |  |  |
| **Perceived ease of use** |  |  |  |  |  |
| U_PEU1 |  |  | .774 |  |  |
| U_PEU2 |  |  | .881 |  |  |
| U_PEU3 |  |  | .886 |  |  |
| U_PEU4 |  |  | .848 |  |  |
| **Hedonic motivation** |  |  |  |  |  |
| U_HED1 | .592 |  |  |  |  |
| U_HED2 | .701 |  |  |  |  |
| U_HED3 | .690 |  |  |  |  |
| **Price value** |  |  |  | .817 |  |
| U_PV1 |  | -.390 |  | .599 |  |
| U_PV2 |  |  |  | .832 |  |
| U_PV3 | .335 |  |  | .751 |  |
| U_PV4 | .317 |  |  | .798 |  |
| **Habit** |  |  |  |  |  |
| U_HAB1 |  |  |  |  | .806 |
| U_HAB2 |  |  |  |  | .759 |
| U_HAB3 |  |  |  |  | .834 |
| U_HAB4 |  |  |  |  | .831 |
| U_HAB5 |  |  |  |  | .855 |
| U_HAB6 |  |  |  |  | .759 |
