# Supplementary material for: Utilizing Health Behavior Change and Technology Acceptance Models to Predict the Adoption of COVID-19 Contact Tracing Apps: Cross-sectional Survey Study
Source: J Med Internet Res. 2021 May 19;23(5):e25447. doi: 10.2196/25447 (PMC8136409; doi:10.2196/25447)
Supplement: Multimedia Appendix 4 [file jmir_v23i5e25447_app4.doc]

**Multimedia Appendix 4**

Supplementary Table S4. Description of a COVID-19 contact tracing app

| German version (original) | English version (translation) |
| --- | --- |
| Für den Umgang mit der COVID-19-Pandemie werden aktuell mehrere Apps entwickelt. Diese Apps sollen unter anderem folgende Funktionen erfüllen:  1. Contact Tracing - das bedeutet Erfassen und Nachzeichnen von Infektionsketten und Bewegungen, sodass Personen gezielt darüber informiert werden können, ob sie Kontakt zu COVID-19-Erkrankten hatten  2. Information und Unterstützung der Bevölkerung im Falle einer Infektion oder bei Kontakt mit einer infizierten Person  3. Unterstützung von Personen in Quarantäne durch Überwachung des aktuellen Gesundheitszustands und Bereitstellung von Gesundheitsinformationen  Deshalb soll in Erfahrung gebracht werden, welche Erwartungen und Erfahrungen Sie mit diesen Apps verbinden und wie Sie die Nutzung und den Nutzen dieser Apps einschätzen. | To address the COVID-19 pandemic, several smartphone apps are currently in development. These apps are supposed to have the following functionalities, among others:   1. Contact Tracing – this means monitoring and tracking of infection chains and movements to inform people if they were in contact with people with COVID-19 infections 2. Inform and support the population in case of an infection or if they get in contact with an infected person 3. Provide support for persons in quarantine by monitoring their current health status, and providing health information   Therefore, we want to capture your expectations and experiences regarding these apps, and we would like you to assess the use and usefulness of these apps. |
