# Supplementary material for: Utilizing Health Behavior Change and Technology Acceptance Models to Predict the Adoption of COVID-19 Contact Tracing Apps: Cross-sectional Survey Study
Source: J Med Internet Res. 2021 May 19;23(5):e25447. doi: 10.2196/25447 (PMC8136409; doi:10.2196/25447)
Supplement: Multimedia Appendix 5 [file jmir_v23i5e25447_app5.doc]

**Multimedia Appendix 5**

Supplementary Table S5. Bivariate correlations (Pearson; point biserial) between sociodemographic data, threat appraisals, additional attitudinal variables (personalization, data privacy concerns), and adoption intentions as well as frequency of use of COVID-19 contact tracing apps

|  | 1 | 2 | 3 | 4 | 5 | 6 | 7 | 8 | 9 | 10 | 11 | 12 | 13 | 14 | 15 | 16 |
| --- | --- | --- | --- | --- | --- | --- | --- | --- | --- | --- | --- | --- | --- | --- | --- | --- |
| 1 Age | 1 |  |  |  |  |  |  |  |  |  |  |  |  |  |  |  |
| 2 Gender (ref. female) | .20^c^ | 1 |  |  |  |  |  |  |  |  |  |  |  |  |  |  |
| 3 Persons per household | -.13^a^ | .06 | 1 |  |  |  |  |  |  |  |  |  |  |  |  |  |
| 4 Education (ref. lower secondary) | -.19^c^ | -.05 | .06 | 1 |  |  |  |  |  |  |  |  |  |  |  |  |
| 5 Income (€501-€1000)^d^ | -.35^c^ | -.13^a^ | -.02 | .01 | 1 |  |  |  |  |  |  |  |  |  |  |  |
| 6 Income (€2501 or higher) | .54^c^ | -.24^c^ | -.01 | .11^a^ | - | 1 |  |  |  |  |  |  |  |  |  |  |
| 7 Region (urban) | -.14^a^ | -.07 | .06 | .08 | .10 | -.09 | 1 |  |  |  |  |  |  |  |  |  |
| 8 Region (metropolitan) | -.05 | .02 | -.10 | .06 | .00 | .05 | - | 1 |  |  |  |  |  |  |  |  |
| 9 Migration background | -.03 | .13^a^ | .12^a^ | -.01 | -.06 | -.12^a^ | -.08 | .11 | 1 |  |  |  |  |  |  |  |
| 10 Perceived susceptibility | -.04 | -.07 | .04 | .08 | .03 | .02 | .03 | -.03 | .00 | 1 |  |  |  |  |  |  |
| 11 Anticipatory anxiety | -.18^b^ | -.09 | .03 | .17^b^ | .04 | -.05 | .02 | .03 | .01 | .46^c^ | 1 |  |  |  |  |  |
| 12 Anticipated emotion | -.03 | -.15^b^ | -.05 | .04 | -.03 | -.08 | -.03 | .08 | .03 | .15^b^ | .45^c^ | 1 |  |  |  |  |
| 13 Data privacy concerns | .20^c^ | .04 | -.17^b^ | -.03 | .00 | .10 | -.09 | .11^a^ | .02 | -.05 | -.04 | .00 | 1 |  |  |  |
| 14 Personalization | -.18^b^ | -.07 | .09 | .12^a^ | .07 | -.08 | .05 | -.03 | .07 | .19^c^ | .38^c^ | .23^c^ | -.27^c^ | 1 |  |  |
| 15 Adoption intentions | -.03 | .04 | .05 | .07 | .00 | -.02 | -.05 | .01 | .08 | .17^b^ | .41^c^ | .24^c^ | -.30^c^ | .57^c^ | 1 |  |
| 16 App use frequency | .10 | .09 | .01 | -.13^a^ | -.09 | .06 | -.16^b^ | -.03 | .02 | .06 | .21^c^ | .09 | -.20^c^ | .17^b^ | .46^c^ | 1 |

^a^ *P<*.05
^b^ *P<*.01
^c^ *P<*.001
^d^ Income is represented by its two anchoring categories (lowest and highest) to improve readability, further results are available upon request from the first author
